# Receptor interacting protein 3-induced RGC-5 cell necroptosis following oxygen glucose deprivation

**DOI:** 10.1186/s12868-015-0187-x

**Published:** 2015-08-04

**Authors:** Wei Ding, Lei Shang, Ju-Fang Huang, Na Li, Dan Chen, Li-Xiang Xue, Kun Xiong

**Affiliations:** Department of Anatomy and Neurobiology, Morphological Sciences Building, School of Basic Medical Sciences, Central South University, 172 Tongzi Po Road, Changsha, 410013 Hunan China; Department of Biochemistry and Molecular Biology, Health Science Center, Peking University, Beijing, 100191 China

**Keywords:** Retinal ganglion cell-5, Receptor-interacting protein 3, Oxygen glucose deprivation, Necroptosis, Oxidative stress

## Abstract

**Background:**

Necroptosis is a type of regulated form of cell death that has been implicated in the pathogenesis of various diseases. Receptor-interacting protein 3 (RIP3), a member of the RIP family of proteins, has been reported as an important necroptotic pathway mediator in regulating a variety of human diseases, such as myocardial ischemia, inflammatory bowel disease, and ischemic brain injury. Our previous study showed that RIP3 was expressed in rat retinal ganglion cells (RGCs), where it was significantly upregulated during the early stage of acute high intraocular pressure. Furthermore, RIP3 expression was co-localized with propidium iodide (PI)-positive staining (necrotic cells). These results suggested that RIP3 up-regulation might be involved in the necrosis of injured RGCs. In this study, we aimed to reveal the possible involvement of RIP3 in oxygen glucose deprivation (OGD)-induced retinal ganglion cell-5 (RGC-5) necroptosis.

**Methods:**

RGC-5 cells were cultured in Dulbecco’s-modified essential medium and necroptosis was induced by 8 h OGD. PI staining and flow cytometry were performed to detect RGC-5 necrosis. RIP3 expression was detected by western blot and flow cytometry was used to detect the effect of RIP3 on RGC-5 necroptosis following OGD in *rip3* knockdown cells. Malondialdehyde (MDA) lipid peroxidation assay was performed to determine the degree of oxidative stress.

**Results:**

PI staining showed that necrosis was present in the early stage of OGD-induced RGC-5 cell death. The presence of RGC-5 necroptosis after OGD was detected by flow cytometry using necrostatin-1, a necroptosis inhibitor. Western blot demonstrated that RIP3 up-regulation may be involved in RGC-5 necroptosis. Flow cytometry revealed that the number of OGD-induced necrotic RGC-5 cells was reduced after *rip3* knockdown. Furthermore, MDA levels in the normal RGC-5 cells were much higher than in the *rip3*-knockdown cells after OGD.

**Conclusions:**

Our findings suggest that RGC-5 cell necroptosis following OGD is mediated by a RIP3-induced increase in oxidative stress.

## Background

Necrosis has been considered as an uncontrollable form of cell death for a long time, which has the morphological features of losing plasma integrity and organelle swelling. Recently, more and more evidences have showed that necrosis can be regulated by TNF-α, Fas ligand or ischemia–reperfusion, etc. The type of regulated form of necrosis termed necroptosis occurs in many cell types. Recent studies have shown the presence of necroptosis in glutamate-induced hippocampal neuronal injury [1, 2], oxygen glucose deprivation (OGD)-induced cortical neuronal damage [3, 4], and hemin-induced glial cell damage [5]. Rosenbaum et al. [6] found necroptotic cells in the retinal ganglion cell layer during acute high intra-ocular pressure (aHIOP). Several recent studies have suggested that receptor interacting protein 3 (RIP3) plays an important role in necroptosis in many cell types. Vieira et al. [7] showed that RIP3 mediated neuronal cell death and its expression was upregulated in primary hippocampal neurons following OGD-induced injury. Wang et al. [8] found that the expression of RIP3 could be suppressed by Necrostatin-1 (Nec-1) in ouabain-induced spiral ganglion neuronal injury. Viringipurampeer et al. [9] showed that morpholino gene knockdown of *rip3* can rescue dying photoreceptors in a zebrafish model of retinal degeneration. Dvoriantchikova et al. [10] also showed that mouse RGC necroptosis might be caused by inflammatory responses induced by RIP3.

Though recent studies showed that mixed lineage kinase domain-like protein (MLKL) is downstream of RIP3 in necroptosis [11], the production of reactive oxygen species (ROS) mediated by the activation of RIPs is probably the most studied and well accepted mechanism. Zhang et al. examined TNF-induced necrosis in NIH 3T3 cells and showed that RIP3 regulates TNF-induced ROS overproduction by activating metabolic enzymes, leading to necrosis via damaging of cellular membranes and organelles [12]. Son et al. [13] showed that Nec-1 can significantly reduce ROS production in Theiler’s murine encephalomyelitis virus (TMEV)-infected macrophages. Our previous studies showed that Timosaponin B-II, an anti-oxidative monomer extracted from *Rhizoma anemarrhenae*, reduced retinal ganglion cell-5 (RGC-5) cell necroptosis by inhibiting ROS accumulation [14]. Together, these results suggested that ROS accumulation might directly lead to necrosis.

In our previous study, we found that necroptosis occurs in RGC-5 at 24 h following elevated hydrostatic pressure (EHP) [15]. Moreover, our study showed that RIP3 was mainly expressed in RGCs in vivo in rats, and the expression of RIP3 is significantly upregulated at the early stage of aHIOP [16]. These results indicated that RIP3 might be involved in the necroptosis of RGCs following injury. Therefore, in our recent study we focused on the involvement of RIP3 during necroptosis. In the meantime, various pathophysiology mechanisms have been proposed to cause RGC damage during aHIOP in vivo, with high pressure being only one aspect [15]. Ischemia–hypoxia of RGCs induced by compression of the central retinal artery following aHIOP is a more important mechanism [17]. Therefore, the model of OGD-induced damage (the classical model in vitro to simulate ischemia–hypoxia) was applied in our present study to detect whether it could induce necroptosis in RGC-5 cells and to determine the role of RIP3 in this process. As mentioned above, ROS were indicated to play a direct role in RIP3-mediated necroptosis, so this was also explored simultaneously. Our study will help gain a better understanding of the mechanism of RGC necrosis in acute hypoxic-ischemic retinal diseases and provide experimental evidence to determine a possible target for inhibiting this process in future translational medicine.

## Methods

### Reagents

Rabbit anti-RIP3 antibody, Nec-1, and propidium iodide (PI) were obtained from Sigma-Aldrich (St Louis, MO, USA), rabbit anti-β-tubulin was from Abcam (Cambridge, UK), and the fluorescein isothiocyanate-Annexin V/PI apoptosis assay kit was from Clontech (Mountain View, CA, USA). Morpholino oligonucleotides were synthesized by Gene Tools, LLC (Philomath, OR, USA). Bicinchoninic acid assay was purchased from Pierce (Rockford, IL, USA). Lipid peroxidation (MDA) was obtained from Jian-Cheng Biotechnical Co. (Nanjing, Jiangsu, China). Goat anti-rabbit secondary antibody was obtained from Jackson Immuno Research Inc. (Lancaster, PA, USA).

### Cell culture

Mouse RGC-5 cells were contributed by the Department of Ophthalmology, Second Hospital of Ji Lin University, China [18]. RGC-5 cells were grown in Dulbecco’s modified Eagles medium (DMEM) (HyClone Laboratories, Inc., Logan, UT, USA) and supplemented with 10% fetal bovine serum (FBS, HyClone Laboratories, Inc.), 100 U/mL penicillin and 100 μg/mL streptomycin (HyClone Laboratories, Inc.). The cells were grown at 37°C under a humidified atmosphere of 5% CO_2_. The RGC-5 cells used in the experiment were with two to three passages post-thawing to minimize variability in the assays based on our observations. The density of RGC-5 cells was around 70% in 6 mL culture media in a 50-mL flask before OGD.

### OGD model and Nec-1 use

When the density of RGC-5 cells was around 70%, cells were washed twice with glucose-free DMEM (Sigma-Aldrich), then placed in the same medium in an anaerobic chamber with 95% N_2_ and 5% CO_2_ for 8 h at 37°C to induce OGD [19, 20]. After 8 h, OGD was terminated by removing the cell culture flasks from the anoxic chamber and replacing the glucose-free DMEM with regular culture medium. The cells were then maintained in a regular 5% CO_2_ incubator to recover for each time point (6 and 12 h).

Nec-1 was dissolved in dimethyl sulfoxide (DMSO) (AppliChem, Gatersleben, Germany) at 1 mg/mL, and cells were pretreated with 10 μM for 24 h before OGD. After 8 h in the hypoxic chamber, OGD was stopped by replacing the medium with regular culture medium containing Nec-1 [21].

### PI staining

At each recovery time point (6 and 12 h), the coverslips were washed in 0.01 M PBS for 3 min, and incubated in 10 μg/mL PI-dye solution at 37°C for 30 min. Subsequently, cells were washed in 0.01 M PBS for 3 min and fixed in 4% paraformaldehyde for 20 min. Then, cells were washed in PBS, counterstained with DAPI, and covered with anti-fading mounting medium before fluorescence microscopy (Nikon, Eclipse 80i, Tokyo, Japan). Motic pathology image analysis software (Motic Inc., Xiamen, China) was used to count cells.

### Western blot

At each survival time point, cells were homogenized on ice in digestion buffer [150 mM NaCl, 25 mM Tris–HCl (pH 7.4), 2 mM EDTA, 1.0% Triton X-100, 1.0% sodium deoxycholate, 0.1% SDS] containing a cocktail of protease inhibitors (Sigma-Aldrich). Then, the homogenates were centrifuged at 10,000×*g* for 20 min at 4°C. The supernatants were collected and the protein concentration was determined using the bicinchoninic acid assay kit. A total of 100 μg of protein in 62.5 mM Tris loading buffer (pH 6.8, containing 25% glycerol, 2% SDS, 0.01% bromophenol blue, and 5% β-mercaptoethanol, Bio-Rad, Hercules, CA, USA) was boiled for 5 min, separated by SDS–polyacrylamide gel electrophoresis and transferred onto a nitrocellulose membrane (Bio-Rad). Non-specific binding was blocked with PBS containing 5% nonfat milk (Bio-Rad) and 3% bovine serum albumin (Sigma-Aldrich) for 1 h. Membranes were incubated with anti-RIP3 (1:200) or anti-β-tubulin (1:1,000) antibodies overnight, washed, and subsequently incubated in HRP-conjugated secondary antibodies (1:20,000, Bio-Rad) for 2 h. Immunoblotting products were visualized with an ECL Plus™ Western Blotting Detection kit according to the manufacturer’s instruction (GE Healthcare Life Sciences, NJ, USA), and images were captured in a Molecular Dynamics Phosphorimager (Nucleo Tech Inc., CA, USA). Western blot bands were measured with Image J (National Institutes of Health, MD, USA) to analyze the optical density (OD). The average OD of RIP3 and β-tubulin were compared, and the average relative value was obtained. Each experiment was repeated at least three times.

### Flow cytometry

The cells attached to flasks were trypsinized, followed by a gentle wash. Cells were resuspended in 200 μL of 1× binding buffer, following which 5 μL of 20 μg/mL AnnexinV and 10 μL of 50 mg/mL PI were added to the suspension, and incubated at room temperature for 15 min in the dark. Cells were then washed and analyzed by FACSCalibur™ (Becton, Dickinson Company, NJ, USA). The percentages of cells in each quadrant were analyzed using ModFit software (Verity Software House, ME, USA). Statistical analyses of flow cytometry results were conducted by calculating the number of PI-positive cells. All tests were repeated three times.

### Knockdown of *rip3* expression using anti-sense morpholinos

The expression of *rip3* was inhibited using anti-sense morpholino oligos, following Garlapati’s described method [22]. The *rip3* morpholino oligo and standard control oligo were designed and purchased from Gene Tools LLC, whereby the *rip3* morpholino oligo used for inhibition of *rip3* translation had the following sequence: 5′-AGGCCATAACTTGACAGAAGACATC-3′. The standard control oligo sequence was as follows: 5′-CCTCTTACCTCAGTTACAATTTATA-3′. When the density of RGC-5 cells was around 80%, the cells were incubated with 1 μM *rip3* morpholino oligo for 48 h.

### Immunofluorescence staining

Coverslips with fixed cells (*rip3*-knockdown and normal control) were washed in 0.01 M PBS for 3 min, incubated in 5% BSA, followed by rabbit anti-RIP3 antibody (1:200) overnight. Then, cells were incubated with Cy3-conjugated donkey anti-rabbit secondary antibodies at 1:200 (Invitrogen, Carlsbad, CA, USA), and covered with an anti-fading mounting medium before microscopic examination (Eclipse 80i, Nikon, Tokyo, Japan).

### PCR analysis

The cells cultured in the flasks were harvested and RNA was isolated using TRIzol reagent (Invitrogen). cDNA was synthesized using Thermoscript (Invitrogen) from 1 μg of total RNA. Each primer pair (β-actin: forward primer 5′-CAACTTGATGTATGAAGGCTTTGGT-3′, reverse primer 5′-ACTTTTATTGGTCTCAAGTCAGTGTACAG-3′; RIP3: forward primer, 5′-GATTTTGGCCTGTCCACGTT-3′, reverse primer 5′-CAGGCCCAACTGATGTGTCC-3′). β-actin was used as the normalization control. The PCR conditions were as follows: 94°C for 3 min, 36 cycles of 94°C for 45 s, 55°C for 50 s, and 72°C for 2 min, with a final extension at 72°C for 10 min.

### MDA concentration assay

ROS levels were measured by MDA assay. At each survival time point, cells were digested by sonication on ice, in digestion buffer containing a cocktail of protease inhibitors. Then, the homogenates were centrifuged at 10,000×*g* for 20 min at 4°C. MDA levels in RGC-5 extracts were assayed using a commercial kit according to the manufacturer’s instruction (Jian-Cheng Biotechnical Co.) as described in our previous study [23]. Equal quantities (100 µg) of supernatant were loaded in each well and each analysis was performed in triplicate.

### Data analysis

One-way analysis of variance was performed to test differences in average values between groups. All results were presented as mean ± standard deviation. A value of *P* < 0.05 was considered statistically significant. The data were analyzed by using SPSS 19.0 (SPSS Inc., Chicago, IL, USA).

## Results

### Necroptosis induction following OGD

**Fig. 1:**
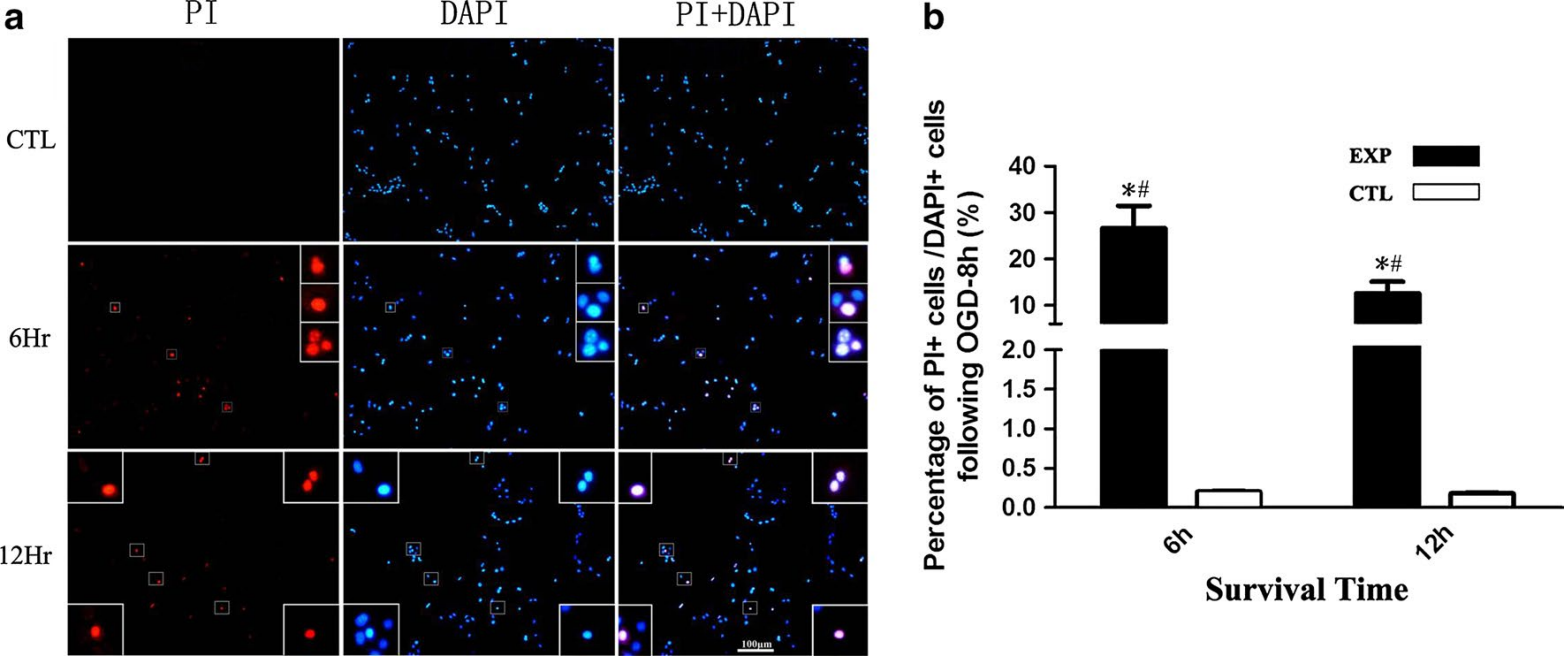
RGC-5 cell necrosis following 8 h OGD. **a** PI/DAPI staining of RGC-5 cells at 6 and 12 h following OGD, the small frame is the enlargement of the indicated area, *bar* 100 μm; *bar* 400 μm in small frame. **b** The statistical analysis of RGC-5 necrotic cells, * vs CTL, *P* < 0.05; *^#^ vs *^#^, *P* < 0.05.

PI staining was used to distinguish necrotic cells from normal ones [24, 25]. PI and DAPI double labeling showed that there was no obvious PI staining in the normal control group (CTL), while PI-positive cells were observed after 6 and 12 h of re-oxygenation following OGD (Fig. 1a). Meanwhile, the number of PI-positive cells after 6 h re-oxygenation was more than after 12 h (*P* < 0.05, Fig. 1b).

**Fig. 2:**
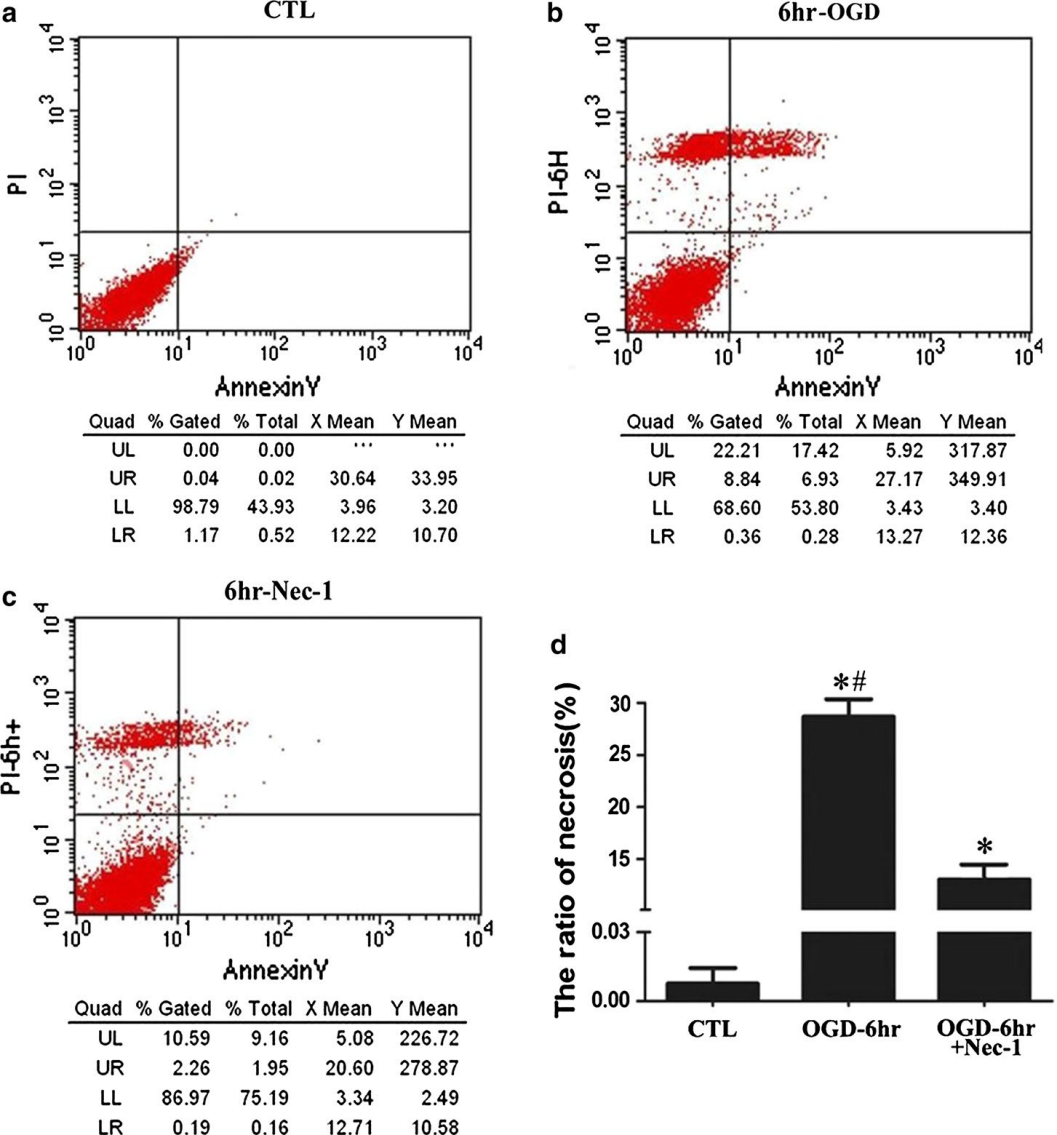
Ratio of necrotic cells is reduced following Nec-1 pre-treatment by OGD. **a** Normal control cells; **b** RGC-5 cell necrosis after OGD; **c** RGC-5 cells were pre-treated with Nec-1 (10 μM) to block necroptosis for 24 h before OGD and analysis of necroptotic cells. Cells were stained with Annexin fluorescein isothiocyanate and PI, and analyzed by FACS using FL1 (Annexin V) and FL3 (PI) channels. **d** The statistical analysis of RGC-5 necrosis, * vs CTL, *P* < 0.05; ^#^ vs Nec-1 pre-treatment, *P* < 0.05.

The immunofluorescence results indicated that there was a large number of necrotic RGC-5 cells after 6 h re-oxygenation following OGD; thus, we chose this time point to analyze cellular necroptosis by flow cytometry with PI/Annexin V double staining following pretreatment with Nec-1 (RGC-5 cells were incubated with 10 µM Nec-1 for 24 h prior to OGD). The results showed that necrosis occurred after OGD (Fig. 2b), but the number of necrotic (PI-positive) cells decreased significantly with Nec-1 pretreatment (Fig. 2c, d, *P* < 0.05). These results indicate that RGC-5 cell necrosis can be inhibited by Nec-1 and that necroptosis occurred at the early stage of OGD.

### RIP3 upregulation following OGD

**Fig. 3:**
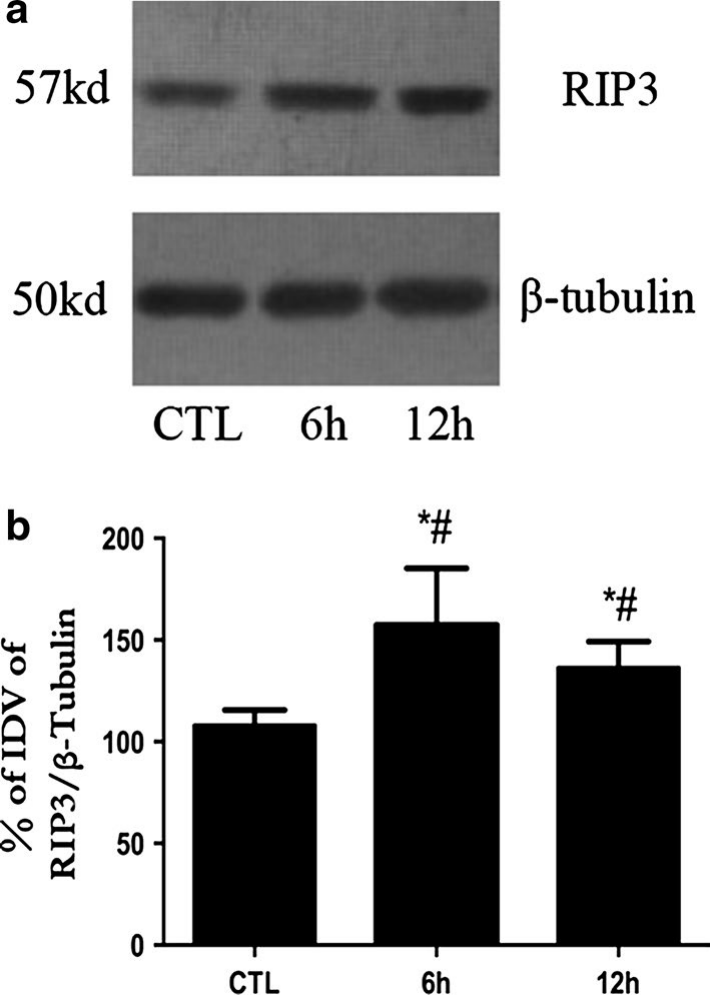
RIP3 protein expression is up-regulated after OGD. **a** Western blot bands of RIP3 and β-tubulin expression; **b** OD analysis of RIP3; *error bars* represent standard deviation, * vs CTL, *P* < 0.05; ^#^ vs ^#^, *P* < 0.05.

The western blot results showed that RIP3 mainly exhibited as a single 57-kDa band in all groups (Fig. 3a). The bands in the 6- and 12-h re-oxygenation groups were thicker than the normal control group after OGD. Statistical analysis of OD values indicated that OGD up-regulated the expression of RIP3 at the early stage (*P* < 0.05, Fig. 3b), with a significantly more intense RIP3 band evident in the 6-h re-oxygenation group.

### Preparation of *rip3*-knockdown RGC-5 cells

**Fig. 4:**
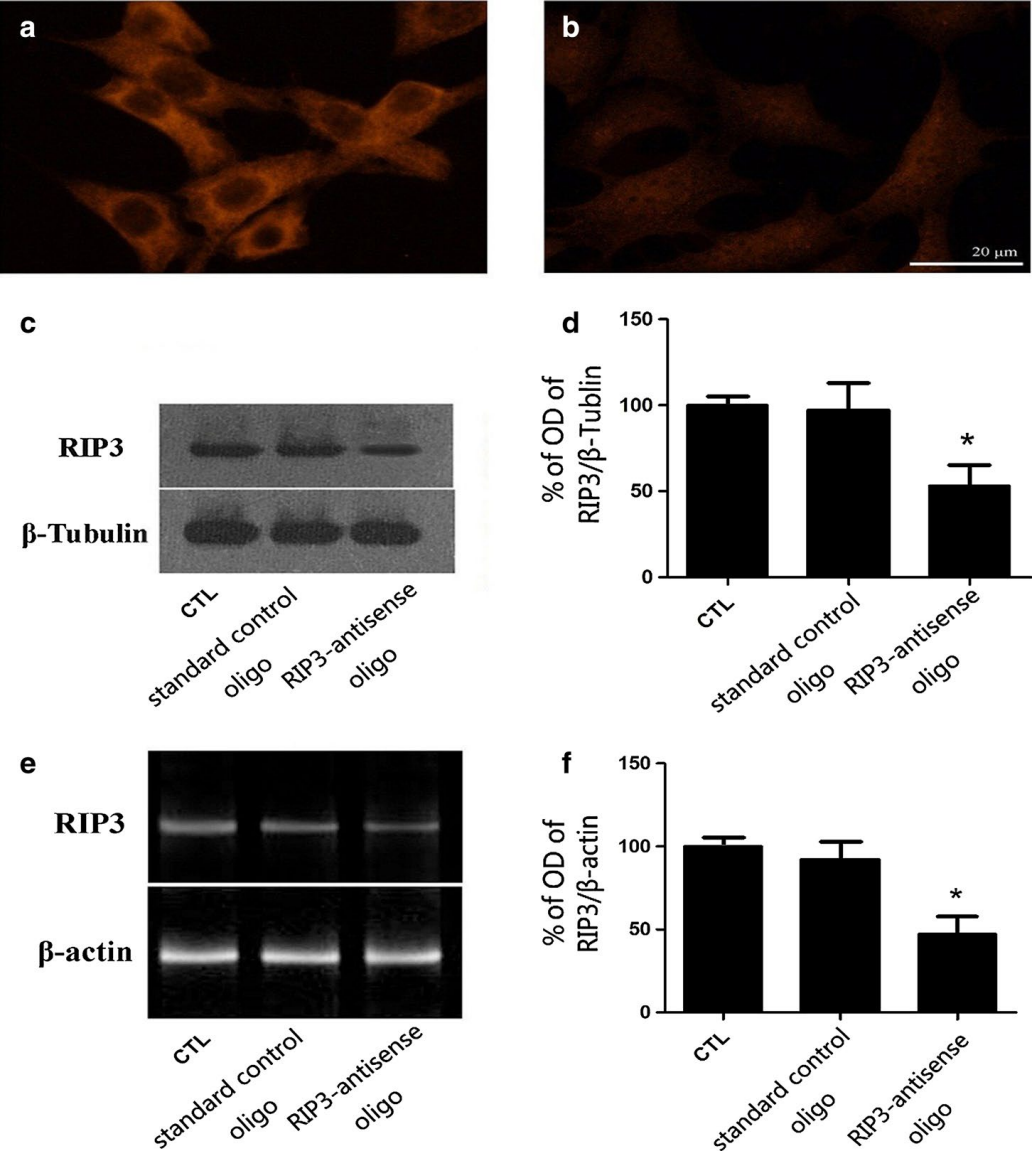
Establishment of RGC-5 *rip3* knockdown cell line. RIP3 immunofluorescence staining of RGC-5 cells after *rip3* knockdown (**a** normal RGC-5; **b** *rip3*-knockdown RGC-5). Western blot results (**c**, **d**). PCR results (**e**, **f**), *bar* 20 μm in **a**, **b**, * vs CTL, *P* < 0.05.

RIP3 immunofluorescence revealed that when RGC-5 cells were treated with 1 μM custom RIP3 antisense morpholino oligos for 48 h, the intensity of fluorescence was weaker than the normal control (Fig. 4a, b). The western blot results showed that compared with normal RGC-5 cells and those treated with standard control oligos, the expression of RIP3 in *rip3*-knockdown cells was reduced (Fig. 4c). Statistical analysis of OD indicated that the difference was significant (*P* < 0.05, Fig. 4d). RT-PCR analysis showed a similar tendency at the mRNA level. (*P* < 0.05, Fig. 4e, f). Together, these results indicate that *rip3* knockdown in RGC-5 cells was successful.

### OGD-induced RGC-5 necroptosis mediated by RIP3

**Fig. 5:**
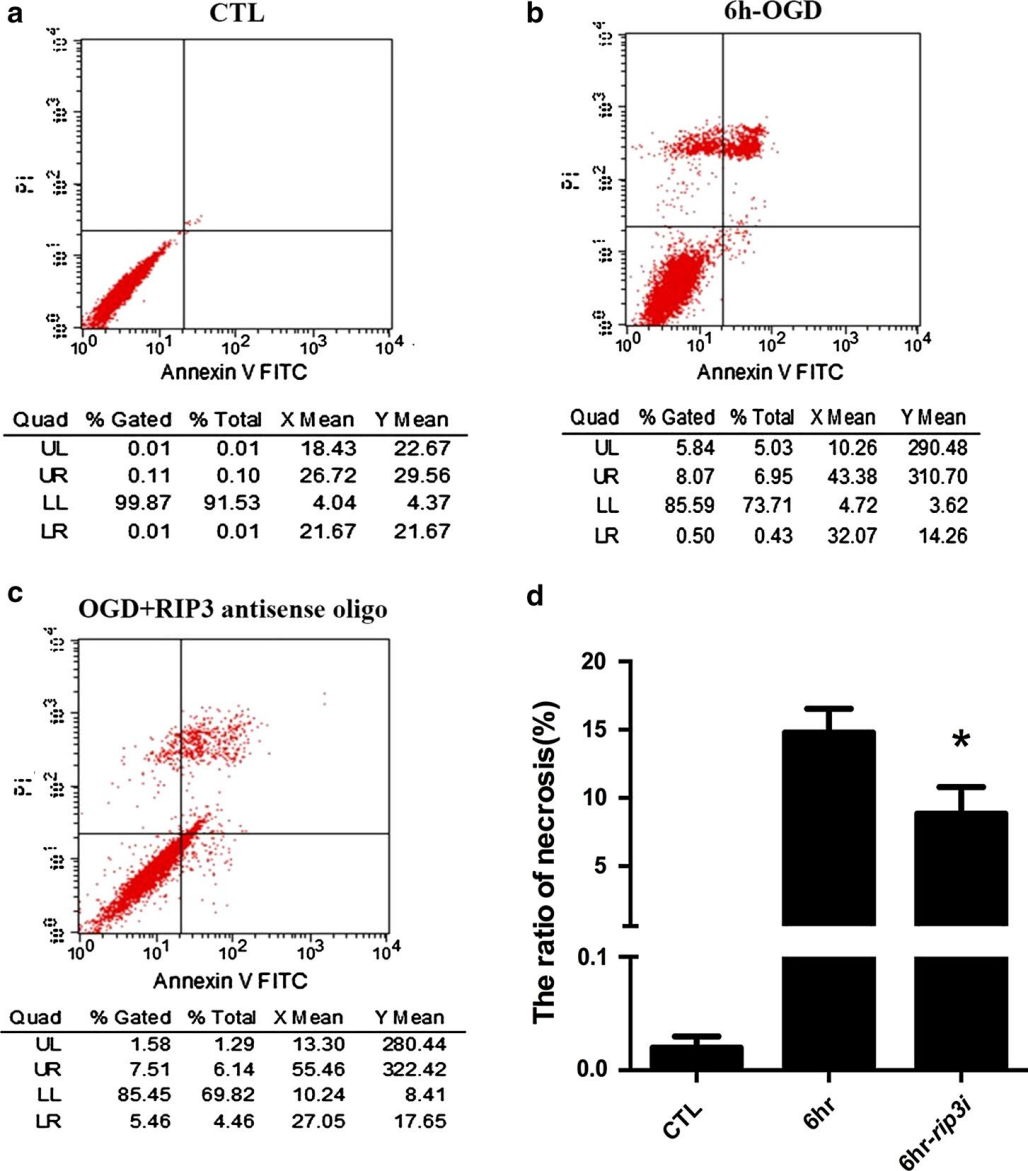
The ratio of necrotic cells decreased following *rip3* knockdown by OGD. **a** Normal RGC-5; **b** normal RGC-5 necrosis after OGD; **c** *rip3*-knockdown RGC-5 necrosis after OGD. **d** The statistical analysis of RGC-5 necrosis, * vs OGD, *P* < 0.05.

Flow cytometry with PI/Annexin V double staining of *rip3*-knockdown and normal RGC-5 cells was applied to determine the involvement of RIP3 in OGD-induced necroptosis after 6 h re-oxygenation. The results showed that there were more necrotic cells in both the normal OGD group and *rip3*-knockdown OGD group compared with the normal control group (Fig. 5a–c). However, the number of PI-positive cells in the *rip3*-knockdown group was decreased significantly compared with the normal OGD group (*P* < 0.05, Fig. 5d).

### MDA levels in *rip3*-knockdown RGC-5 cells decreased following OGD

**Fig. 6:**
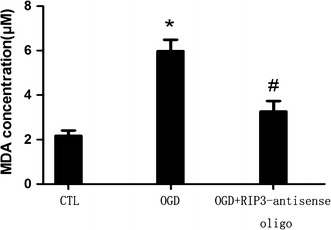
MDA is reduced following *rip3* knockdown by OGD insult. * vs ^#^ *P* < 0.05; ^#^ vs CTL, *P* < 0.05.

The MDA concentration assay showed that after 6 h re-oxygenation, MDA levels in the normal OGD group and the *rip3*-knockdown OGD group increased significantly compared with the normal control group, but the level of MDA in the *rip3*-knockdown OGD group decreased significantly compared with the normal OGD group (*P* < 0.05, Fig. 6).

## Discussion

As a form of regulated cell death, necroptosis has attracted widespread attention in recent years. A growing number of studies have confirmed the presence of necroptosis in many diseases. Necroptosis has been indicated in injured cells in tumor necrosis factor (TNF)-induced murine fibrosarcoma L929 cells [26], TNF-induced tubular epithelial cells of donor kidneys [27], pathogenic free-living *Naegleria fowleri*-induced Jurkat T cells [28], TNF-related apoptosis-inducing ligand (TRAIL)-induced HepG2 cells [29], and acetaminophen-induced acute liver failure, to name a few [30]. Necroptosis was first found in non-neuronal cells; however, recent studies indicated it could also occur in neurons. Studies showed that in rat spinal cord injury, Nec-1 could protect neurons and improve physiological function at the early stage [31]. Dai et al. [32] revealed that necroptosis occurred in primary cortical neurons, which were injured by ferrous chloride. Li et al. [33] reported that 2 h after exposure to *N*-methyl-d-aspartic acid (NMDA), necroptosis occurred in cultured cortical neurons. Rosenbaum et al. [6] found a number of necroptotic cells in the rat retinal ganglion cell layer at 6 h after aHIOP. Our previous studies also indicated that necroptosis occurred in RGC-5 cells at an early stage following elevated hydrostatic pressure in vitro [15] or 300 μM hydrogen peroxide (H_2_O_2_) treatment [14]. Recently, in vitro models of OGD have been widely applied to simulate neuronal ischemia in vivo. Wang et al. [34] reported that the OGD model (6 h OGD followed by 24 h re-oxygenation) in cultured cortical neurons mimicked cerebral ischemia, and Tasca et al. [35] induced OGD in primary neurons to mimic the cellular death observed in models of brain ischemia in vivo. Therefore, we chose the OGD model in this study and the data indicated that necrosis occurred following 8 h OGD, with the number of necrotic cells increasing obviously after 6 and 12 h re-oxygenation. Flow cytometry also detected more necrotic cells at 6 h after re-oxygenation. More importantly, the number of necrotic cells significantly reduced following pretreatment with Nec-1. Together, these results suggest that necroptosis might be a form of cell death that widely exists in injured cells.

Many scientists have investigated the regulatory mechanism of necroptosis. Many different molecules were considered to participate in the occurrence and regulation of necroptosis, such as HtrA2/Omi, ubiquitin C-terminal hydrolase (UCH-L1) [36], CDGSH iron-sulfur domain-containing protein 1 (CISD1) [37], and calpain [38]. Of the numerous pathways investigated for their involvement in necroptosis, the RIP signaling pathway was the one that was first studied and gained the most attention. For instance, previous studies indicated that the expression of RIP1 was upregulated in OGD-induced neuronal damage and mediated necroptosis [3]. Roychowdhury et al. [39] revealed that RIP3-driven necroptosis was a key step of ethanol-induced hepatocyte injury. A recent study showed that RIP3 interacted with RIP1 via the RIP homotypic interaction motif (RHIM), when apoptosis was interrupted and, therefore, induced necroptosis by direct or indirect phosphorylation [40]. Phosphorylation of RIP3 promoted the phosphorylation of RIP1, which activated key enzymes of metabolic pathways and increased ROS production, thereby inducing necroptosis [41]. More recently, a study showed that following mutation of *rip3*, carbobenzoxy-valyl-alanyl-aspartyl-[*O*-methyl]-fluoromethylketone (zVAD) inhibited TNF-α induced apoptosis, but did not promote necroptosis, Therefore, RIP3 was the key target regulating necroptosis [12]. Our previous study indicated that the expression of RIP3 was upregulated at the early stage of aHIOP [16]. Thus, in this study, we focused on confirming the involvement of RIP3 in OGD-induced RGC-5 necroptosis. The western blot results showed that after 8 h OGD and 6 or 12 h re-oxygenation, RIP3 expression was significantly upregulated, suggesting that the RIP3 upregulation may be related to OGD-induced necroptosis. When *rip*3 was knocked down using morpholino oligos, flow cytometry results showed that the number of necrotic cells was significantly reduced compared with the normal OGD group. Again, these results suggest that RIP3 is a key molecule mediating OGD-induced RGC-5 cell necroptosis. Furthermore, we observed that the MDA concentration, which describes the degree of oxidant stress, increased significantly after OGD compared with the normal control. Furthermore, MDA concentration decreased significantly in *rip3*-knockdown cells compared with normal controls following OGD. According to the data described herein, we speculated that OGD-induced RGC-5 necroptosis might be caused by RIP3 upregulation-mediated ROS accumulation resulting in necrosis. Shindo et al. [42] also reported similar results, whereby RIP-mediated necroptosis was associated with ROS accumulation in mouse embryo fibroblasts (MEFs).

Though RIP3 was shown to regulate OGD-induced RGC-5 necroptosis, the flow cytometry results showed that it was not completely inhibited when the expression of RIP3 was inhibited. Furthermore, the level of MDA was higher in *rip3*-knockdown RGC-5 OGD group compared with normal control group. We speculated that there are three reasons that may account for this phenomenon. First, there might exist other necroptosis-independent forms of cell death responsible for RGC-5 cell necrosis. Second, although RIP3 was inhibited by morpholino oligo, the expression of RIP3 was not completely inhibited, therefore the remaining RIP3 could still partially maintain function. Third, many other molecules, such as calpain, HtrA2/Omi, UCH-L1, and CISD1, could be involved in the regulation of necroptosis, whereby the RIP3 signaling pathway would not be the unique regulatory mechanism.

It should be noted that our results show that changes in the ratio in upper left quadrants in the Nec-1 pretreatment group were less than *rip3* knockdown group following OGD, whereas changes in upper right quadrants in the Nec-1 pretreatment group were more than the *rip3* knockdown group following OGD. Based on Ormerod’s analysis of flow cytometry results [43], the upper left (UL) quadrant contains dead cells or cell fragments, the upper right (UR) quadrant contains necrotic or late apoptotic cells. Moreover, in vivo aHIOP [44, 45] or in vitro OGD [46] analyses revealed that the majority of RGC deaths were necrotic like at the early stage (3, 4, or 12 h) of retinal ischemia, whereas apoptosis occurs at 24 h or later after ischemia. Therefore, we speculated that the protective effect of Nec-1 pretreatment was not as effective as *rip3* knockdown in strongly necrotic cells after OGD and 6 h re-oxygenation. However, further research is required to confirm these findings.

In conclusion, our results indicate that RIP3 induces RGC-5 cell necroptosis following OGD via ROS accumulation. Our research may help find a suitable biological target, such as RIP3 or ROS, to prevent RGC necroptosis.
